# Identification of Six Potential Therapeutic Targets Common to Ischemic Stroke and Vascular Dementia: Genetic Insights From an Integrated Bioinformatics Analysis

**DOI:** 10.1002/brb3.71096

**Published:** 2025-11-25

**Authors:** Jian Lyu, Fa Lin, Yi Liu, Yan Chai, Xiaolin Chen, Min Wang, Fumei Liu, Haiyan Xiao, Guibo Sun, Yanming Xie

**Affiliations:** ^1^ NMPA Key Laboratory for Clinical Research and Evaluation of Traditional Chinese Medicine & National Clinical Research Center for Chinese Medicine Cardiology, XiYuan Hospital China Academy of Chinese Medical Sciences Beijing China; ^2^ Department of Neurosurgery, Beijing Tiantan Hospital Capital Medical University Beijing China; ^3^ China National Clinical Research Center For Neurological Diseases Beijing China; ^4^ Institute of Basic Research in Clinical Medicine China Academy of Chinese Medical Sciences Beijing China; ^5^ Department of Epidemiology University of California Los Angeles California USA; ^6^ Institute of Medicinal Plant Development Peking Union Medical College and Chinese Academy of Medical Sciences Beijing China

**Keywords:** integrated bioinformatics analyses, ischemic stroke, Mendelian randomization, therapeutic targets, vascular dementia

## Abstract

**Background:**

The aim was to investigate the potential therapeutic targets for ischemic stroke (IS) and vascular dementia (VD).

**Methods:**

We assessed the causal effects of 2943 plasma proteins on IS and VD using a two‐sample Mendelian randomization (MR) framework. Results were validated via summary data‐based MR (SMR) analysis. A two‐step mediation MR analysis was conducted to elucidate potential causal mechanisms by which plasma proteins influence IS and VD through risk factors. We performed a phenome‐wide association study (PheWAS) MR analysis to explore side effects and additional indications for IS and VD‐associated plasma proteins. We established middle cerebral artery occlusion and reperfusion (MCAO/R) and VD rat models to verify potential therapeutic targets for IS and VD.

**Results:**

Genetically predicted plasma levels of six proteins demonstrated causal relationships with both IS and VD. SMR analysis validated four of these proteins (CD40, F11, F2, and Furin). Atrial fibrillation (AF), type 2 diabetes (T2D), low‐density lipoprotein (LDL), and diastolic blood pressure (DBP) were causally associated with the risk of IS and VD, indicating common risk factors. CD40 and Furin associations with IS and VD appeared to be mediated by AF or DBP. The mechanisms of action of IS‐ and VD‐related proteins primarily involve the complement and coagulation cascade. In vivo experiments confirmed that CD40, Furin, F11, and integrin alpha‐V (ITGAV) were causally associated with IS and VD.

**Conclusions:**

Our findings illuminate causal pathways and potential therapeutic targets for IS and VD. We identified six plasma proteins with causal relationships to IS, VD, and their risk factors, providing insights into using these proteins as therapeutic targets for IS and VD.

## Graphical abstract

This graphical abstract summarizes a multi‐layered analytical framework integrating cis‐eQTLs, UKB‐PPP proteomics, and multivariable Mendelian randomization to dissect the causal links between circulating proteins, vascular risk factors, ischemic stroke (IS), and vascular dementia (VD). After applying stringent selection criteria for instrumental variables, primary MR analyses identified six genetically predicted plasma proteins (CD40, Furin, CD300LF, F11, F2, and ITGAV) with significant causal effects on both IS and VD. Follow‐up PheWAS and transcriptomic analyses further validated protein‐specific phenotypic associations and molecular pathways. Finally, in vivo experiments confirmed the causal influence of CD40, Furin, F11, and ITGAV on IS and VD, supporting these proteins as potential therapeutic targets for preventing brainvascular multimorbidity.

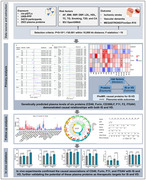

## 1 Introduction

Ischemic stroke (IS) is a neurological disorder caused by disruptions in cerebral blood circulation, characterized by high incidence, disability, and mortality rates (Tsao et al. 2022). According to the latest GBD 2021 mortality data, stroke remains a leading cause of death, with IS accounting for about 70%–80% of cases (GBD 2021 Diseases and Injuries Collaborators 2024; Saini et al. 2021; Roth et al. 2020). The increasing deaths due to IS continue to escalate the global economic burden (Wang et al. 2022; GBD 2019 Stroke Collaborators 2021; Ding et al. 2022). Vascular dementia (VD) is severe cognitive impairment resulting from cerebrovascular diseases, with IS being a major causative factor (Sexton et al. 2019). Following IS, reperfusion injury and blood supply disruptions lead to neurological damage and dysfunctions in motor, cognitive, and language abilities (Iadecola et al. 2019). Particularly with concurrent cerebral arteriosclerosis, reduced blood flow and neuronal oxygen supply cause ischemic necrosis and neurological decline, inducing VD (Wang, Zhang et al. 2020b). VD after IS primarily manifests as a progressive decline in higher cortical functions, with alterations in brain tissue volume disrupting cortical and subcortical connections, causing cerebrospinal fluid (CSF) circulation disorders and neurotransmitter changes, thus impairing dominant hemisphere brain functions (Sun et al. 2022). Research indicates that VD is preventable, and early intervention can significantly reduce its incidence (Pendlebury et al. 2019).

Multimorbidity refers to the simultaneous coexistence of two or more chronic noncommunicable diseases (Langenberg et al. 2023). In 2008, the World Health Organization defined multimorbidity as the presence of at least two chronic diseases within the same patient, forming the basis for many related studies (World Health Organization 2008). A study involving over 2 million people showed high rates of multimorbidity in middle‐aged and elderly populations in China, at 51.6% and 81.3%, respectively (Wang, Yao et al. 2020a). IS combined with VD (IS and VD multimorbidity) is common among the elderly, with VD incidence following IS ranging from 18.2% to 34.2% (Wolters and Ikram 2019). Among those over 65 years old, VD prevalence is 1.50% (Jia et al. 2014). IS and VD multimorbidity poses significant psychological and economic burdens globally. Thrombolysis and other procedures that recanalize cerebral blood vessels often exacerbate inflammatory damage to ischemic brain tissue, sometimes causing irreversible cerebral ischemia reperfusion injury (CIRI) (Wu et al. 2018). Most drug trials for VD overlap with those targeting functional outcomes in cerebrovascular diseases, and no widely accepted treatment regimen for VD exists (Linh et al. 2022). As a prevalent condition among the elderly, IS and VD multimorbidity still has many unmet treatment needs.

In the context of IS and VD multimorbidity, changes in some biomarkers usually precede clinical symptoms during ischemic and hypoxic damage to nerve cells. Identifying biomarkers with high sensitivity, specificity, and accuracy is crucial for clinical precision medicine, personalized prevention, and intervention of IS and VD multimorbidity. As VD develops on the foundation of cerebrovascular disease, any risk factor that directly or indirectly causes cerebrovascular disease can also be a risk factor for VD. Atrial fibrillation (AF), body mass index (BMI), smoking, blood pressure, type 2 diabetes (T2D), coronary atherosclerosis (CA), and blood lipids are the main intervention factors for preventing IS and VD (Report on Stroke Prevention and Treatment in China Writing Group 2020, 2023; Boehme et al. 2017; Chang and Chang 2022). Revealing the potential genetic nature of these risk factors can more precisely identify the pathogenic genetic risk factors, diagnostic markers, and therapeutic targets for IS and VD.

Mendelian randomization (MR) uses genetic variation as a tool to study causal relationships between exposure factors and disease outcomes, utilizing genome‐wide association studies (GWAS) databases (Malik et al. 2018). Due to the random and constant allocation of genetic variation, MR studies are not affected by confounding factors and reverse causality (Verbanck et al. 2018). A 2023 *Nature* study analyzed the plasma proteomic characteristics of 54,219 UK Biobank participants (Sun et al. 2023). The study conducted a comprehensive protein quantitative trait loci (pQTL) mapping for 2923 proteins, identifying 14,287 major genetic associations. This updated the characterization of the plasma proteome's genetic architecture helps elucidate the biological mechanisms behind proteogenomic discoveries, providing opportunities for developing biomarkers, predictive models, and therapeutic targets for IS and VD.

To systematically study the causal pathways and potential targets of human plasma proteins related to IS and VD, we conducted a two‐sample MR analysis to evaluate the causal effects of 2943 plasma proteins on IS and VD, respectively. These results were validated using summary‐data‐based MR (SMR) analysis. Further analysis of the causal relationships between common clinical risk factors and IS and VD identified common risk factors. A two‐step mediation MR analysis based on common risk factors revealed the potential causal mechanisms by which plasma proteins influence IS and VD through these risk factors. We conducted a PheWAS MR analysis to reveal the roles of plasma proteins with causal relationships to both IS and VD across the phenotypic spectrum (Denny et al. 2013). On the basis of MR analysis results, we performed integrated bioinformatics analyses using human transcriptome data to study the mechanisms of action of IS‐ and VD‐related proteins. Through this systematic study, we aim to identify potential therapeutic targets for IS and VD.

## 2 Methods

### 2.1 Study and Overview

In this study, we conducted two‐sample MR analyses to investigate the causal relationships between 2943 plasma proteins and IS and VD, thereby identifying proteins related to IS and VD. We validated these results using SMR analysis. Through a two‐step mediation MR analysis, we explored the potential causal mechanisms by which plasma proteins influence IS and VD via 11 risk factors. Additionally, we conducted PheWAS‐MR analyses to broadly examine plasma proteins with causal relationships to both IS and VD, identifying their potential side effects and roles across the phenotypic spectrum.

**FIGURE 1:**
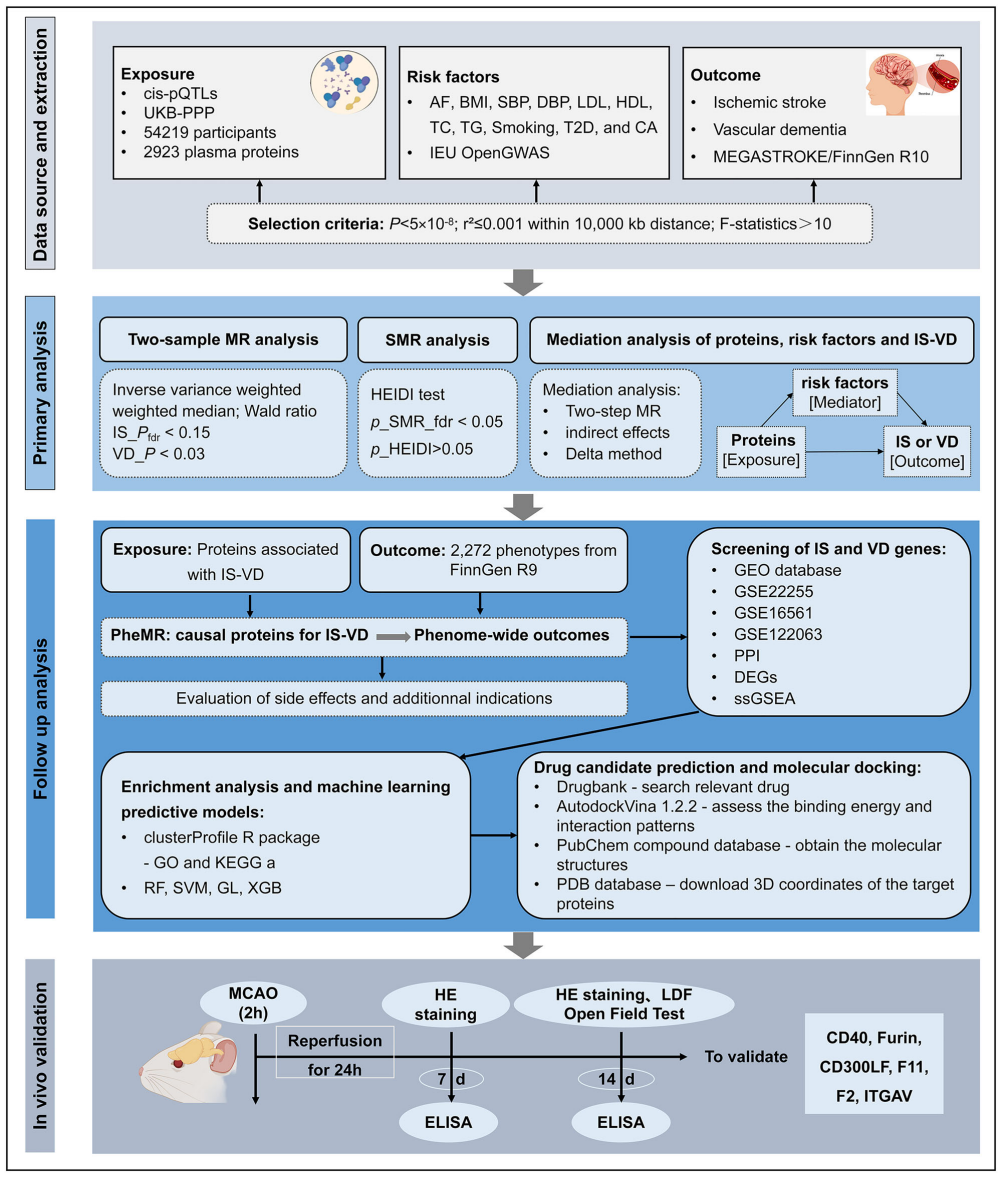
**Overall study design**. AF, atrial fibrillation; BMI, body mass index; CA, coronary atherosclerosis; DBP, diastolic blood pressure; DEGs, differentially expressed genes; GEO, gene expression omnibus; GL, generalized linear; HDL, high‐density lipoprotein; HEIDI, heterogeneity in dependent instruments; IS, ischemic stroke; LDL, low‐density lipoprotein; MR, Mendelian randomization; PPI, protein–protein interaction; RF, random forest; SBP, systolic blood pressure; SMR, summary‐data‐based MR; ssGSEA, Single‐Sample Gene Set Enrichment Analysis; SVM, support vector machine; T2D, type 2 diabetes; TC, total cholesterol; TG, triglycerides; VD, vascular dementia; XGB, extreme gradient boosting.

We performed protein–protein interaction (PPI) analysis on IS‐ and VD‐related proteins obtained from MR analysis, screening for interacting proteins. We utilized IS and VD datasets from the Gene Expression Omnibus (GEO) database to conduct differential expression analysis, correlation analysis, immune infiltration analysis, and enrichment analysis on genes corresponding to PPI‐interacting proteins. Using machine learning methods, we identified feature genes related to IS and VD, predicting the onset of IS and VD based on transcriptome evidence. Finally, we identified potential therapeutic drugs that can form stable structures through the screened feature genes. The overall study design is shown in Figure 1.

### 2.2 Data Source

The summarized data on plasma proteins were sourced from the UK Biobank Pharma Proteomics Project (UKB‐PPP), which conducted proteomic analyses on plasma samples from 54,219 participants and performed comprehensive pQTL mapping for 2923 unique proteins (Sun et al. 2023). IS‐GWAS and VD‐GWAS were selected from the International Stroke Genetics Consortium (Malik et al. 2018) and FinnGen, respectively. On the basis of previous research reports (Report on Stroke Prevention and Treatment in China Writing Group 2020, 2023; Boehme et al. 2017; Chang and Chang 2022), we selected 11 common clinical risk factors, including AF, BMI, systolic blood pressure (SBP), diastolic blood pressure (DBP), low‐density lipoprotein (LDL), high‐density lipoprotein (HDL), total cholesterol (TC), triglycerides (TG), smoking, T2D, and CA, for mediation MR analysis (Roselli et al. 2018; Yengo et al. 2018; Liu et al. 2019; Evangelou et al. 2018; Xue et al. 2018; Willer et al. 2013). The transcriptomic data (ID: GSE22255; GSE16561; GSE122063) were obtained from the GEO database (Krug et al. 2012; Barr et al. 2010; McKay et al. 2019). The detailed data sources used in this study are summarized in Table 1.

**TABLE 1:** Data sources in the current study.

Phenotype	Sample size^a^	Imputation reference panel	Ancestry	Source
Plasma proteome				
Human plasma proteins (*cis*‐pQTLs)	54,219	1000 Genomes phase 3	European	Sun et al. 2023
Ischemic stroke outcomes				
Any ischemic stroke (AIS)	34,217/406,111	1000 Genomes phase 1	European	17 studies (Malik et al. 2018)
Large artery stroke (LAS)	4373/406,111	1000 Genomes phase 1	European	17 studies (Malik et al. 2018)
Cardioembolic stroke (CES)	7193/406,111	1000 Genomes phase 1	European	17 studies (Malik et al. 2018)
Small vessel stroke (SVS)	5386/406,111	1000 Genomes phase 1	European	17 studies (Malik et al. 2018)
Vascular dementia outcomes				
Vascular dementia (VD)	2717/392,463	SISu reference panel	European	FinnGen R10 (https://r10.finngen.fi/)
Vascular dementia‐multiple infarctions (VD‐MI)	525/392,463	SISu reference panel	European	FinnGen R10 (https://r10.finngen.fi/)
Vascular dementia‐mixed (VD‐MX)	363/392,463	SISu reference panel	European	FinnGen R10 (https://r10.finngen.fi/)
Vascular dementia‐subcortical (VD‐SC)	735/392,463	SISu reference panel	European	FinnGen R10 (https://r10.finngen.fi/)
Vascular dementia‐sudden onset (VD‐SO)	157/392,463	SISu reference panel	European	FinnGen R10 (https://r10.finngen.fi/)
Vascular dementia‐other (VD‐O)	142/392,463	SISu reference panel	European	FinnGen R10 (https://r10.finngen.fi/)
Vascular dementia‐undefined (VD‐U)	1256/392,463	SISu reference panel	European	FinnGen R10 (https://r10.finngen.fi/)
Risk factors				
Atrial fibrillation	60,620/1,030,836	HRC	European	IEU Open GWAS (Roselli et al. 2018)
Body mass index	681,275	HRC	European	IEU Open GWAS (Yengo et al. 2018)
Smoking	341,427	HRC	European	IEU Open GWAS (Liu et al. 2019)
Systolic blood pressure	757,601	HRC	European	IEU Open GWAS (Evangelou et al. 2018)
Diastolic blood pressure	757,601	HRC	European	IEU Open GWAS (Evangelou et al. 2018)
Type 2 diabetes	62,892/655,666	HRC	European	IEU Open GWAS (Xue et al. 2018)
Low‐density lipoprotein	173,082	HRC	European	IEU Open GWAS (Willer et al. 2013)
High‐density lipoprotein	187,167	HRC	European	IEU Open GWAS (Willer et al. 2013)
Total cholesterol	187,365	HRC	European	IEU Open GWAS (Willer et al. 2013)
Triglyceride	177,861	HRC	European	IEU Open GWAS (Willer et al. 2013)
Coronary atherosclerosis	51,589/343,079	HRC	European	FinnGen R10 (https://r10.finngen.fi/)
PheWAS				
2272 Phenotypes	412,181	SISu reference panel	European	FinnGen R9 (https://r9.finngen.fi/)
GEO database				
GSE22255 (IS patients vs. controls)	20/20		European	Krug et al. 2012
GSE16561 (IS patients vs. controls)	39/24		European	Barr et al. 2010
GSE122063 (VD patients vs. controls)	8/11		European	McKay et al. 2019

Abbreviations: GEO, gene expression omnibus; GWAS, genome‐wide association studies; IS, ischemic stroke; PheWAS, phenome‐wide association study; pQTLs, protein quantitative trait loci.

^a^ Sample size is shown as a total number for quantitative traits and cases/controls for binary traits.

### 2.3 Two‐Sample MR Analysis of Plasma Proteins With IS and VD

We extracted *cis*‐pQTLs for 2943 proteins from the UKB‐PPP database and selected instrumental variables (IVs) on the basis of the following criteria: (a) single nucleotide polymorphisms (SNPs) within ±1 Mb of the gene region; (b) SNPs with a high independent genetic correlation with plasma proteins (*p* < 5 × 10^−8^, linkage disequilibrium *r* ^2^ < 0.001, genetic windows of 10,000 kb); (c) SNPs with an *F* value less than 10 were excluded. Several estimation approaches were applied in the MR analysis, including inverse‐variance weighted (IVW), Wald ratio, weighted median, MR‐egger, and simple median to estimate the causal relationships. Cochran's *Q* statistic was used to detect heterogeneity in the IVW method; if heterogeneity was present among the IVs, a random effects model was used; otherwise, a fixed effects model was employed (Burgess et al. 2013). Plasma proteins related to IS were selected on the basis of an FDR‐adjusted *p* < 0.15 and pleiotropy >0.05; plasma proteins related to VD were selected on the basis of *p* < 0.03.

### 2.4 SMR Analysis of Positive Plasma Proteins Associated With IS and VD

For the positive plasma proteins related to IS and VD identified through MR, we used the SMR analysis software provided by Yang Lab to detect pleiotropic associations between protein expression levels and IS and VD. This further evaluated the robustness of the identified results using different methods (Zhu et al. 2016). Subsequently, the heterogeneity in dependent instruments (HEIDI) method was used to test whether the associations identified by the SMR test were due to pleiotropy. SMR analysis was performed using version 1.03 of the SMR software (https://cnsgenomics.com/software/smr/#Overview) with default parameters recommended by the developers. A *p* value less than 0.05 was considered statistically significant.

### 2.5 Mediation Analysis of Plasma Proteins, Risk Factors, and IS and VD

To elucidate the potential mechanisms, we conducted mediation analysis using two‐step MR (Carter et al. 2021). First, we explored the causal association between proteins and risk factors through two‐sample MR analysis (beta1). Then, we performed MR analysis on statistically significant associations between risk factors and outcomes (beta2). The indirect effects mediated by risk factors (mediated effect) were calculated by *β*_1_ × *β*_2_. The delta method was used to assess the significance of the mediation effect. Additionally, the percentage of the mediation effect was calculated by dividing the mediation effect by the total effect (primary MR analysis).

### 2.6 MR Analysis of IS‐ and VD‐Related Plasma Proteins Based on PheWAS

PheWAS examines associations between all phenotypes across the phenome and a specific SNP or phenotype. We conducted PheWAS‐MR analysis using plasma proteins positively associated with IS and VD, which also have mediation effects, with summarized data of 2272 phenotypes from the Finnish database. By performing PheWAS‐MR analysis on a series of diseases, we expanded the exploration of side effects of proteins related to four IS subtypes and seven VD subtypes. If the effect direction of additional indications aligns with that of IS and VD, then the identified proteins for treating IS and VD might also be beneficial for the additional indications. A *p*‐FDR value below 0.05 was considered statistically significant.

### 2.7 In Vivo Validation

The middle cerebral artery occlusion and reperfusion (MCAO/R) rat model was established to simulate cerebral ischemia. The Animal Ethical and Welfare Committee approved the animal use protocol (Approval No. SLXD‐20240718022) on July 15, 2024. Following the successful establishment of the MCAO/R model, open field testing was performed on Day 14 to evaluate the achievement of VD status. Laser Doppler flowmetry (LDF) was employed to assess cerebral blood flow distribution in rats on Day 14. Hematoxylin‐eosin (HE) staining was conducted to examine pathological morphology. Serum concentrations of CD40, Furin, CD300LF, F11, F2, and integrin alpha‐V (ITGAV) were quantified using rat‐specific ELISA kits on Days 7 and 14 post‐modeling. Absorbance measurements for each well were obtained via a microplate reader, with subsequent calculations of protein concentrations based on standard curves.

### 2.8 PPI and Expression Analysis of IS‐ and VD‐Associated Plasma Proteins and Their Corresponding Genes

We utilized the STRING database (https://string‐db.org/) to input plasma proteins associated with IS and VD, specifying the species as “Homo sapiens” and setting the medium confidence level to 0.4. PPI analysis was then conducted. Unconnected nodes were excluded from the network, retaining only the interconnected nodes. These nodes were considered proteins with mutual regulatory relationships pertinent to IS and VD.

Gene symbol annotation correction was performed on the GEO data to obtain the expression levels of genes corresponding to IS‐ and VD‐associated proteins for each sample. Differential expression analysis of these genes between normal and IS groups, as well as normal and VD groups, was conducted using R packages. The results were visualized in a heatmap and defined as differentially expressed genes (DEGs). Correlation coefficients for each DEG were calculated using the “cor” command to determine the relationships between each pair of DEGs, and the results were visualized.

### 2.9 Immune Cell Analysis and Enrichment Analysis of DEGs in IS and VD Samples

Using the CIBERSORT command in R, we performed 1000 simulations to obtain the relative content of immune cells for each IS and VD sample (Newman et al. 2015). The results were visualized using bar plots. Single‐Sample Gene Set Enrichment Analysis (ssGSEA) was then conducted to compare the differences in immune cell content between the normal group and the IS group, and between the normal group and the VD group, visualizing the results with box plots. The correlation between DEGs and immune cells, based on ssGSEA scores, was visualized using a clustered heatmap. Gene ontology (GO) enrichment analysis for DEGs was conducted, focusing on biological processes (BP), molecular functions (MF), and cellular components (CC). Additionally, Kyoto Encyclopedia of Genes and Genomes (KEGG) pathway enrichment analysis was performed using the R packages “clusterProfiler” and “enrichplot,” with a significance threshold set at *p* value <0.05.

### 2.10 Machine Learning Risk Models and Nomogram Selection for IS and VD

Four predictive models were constructed using the expression data of DEGs: random forest (RF), support vector machine (SVM), generalized linear (GL), and extreme gradient boosting (XGB) models. Prediction functions were defined, and results for the four models were calculated. To screen for characteristic genes among the DEGs, we generated inverse cumulative distribution plots of residuals, residual box plots, and receiver operating characteristic (ROC) curves for comprehensive evaluation. After selecting the best model, a nomogram was constructed using the characteristic genes and their expression levels in the normal group and the IS or VD group. Decision curves and calibration curves were then constructed to evaluate the accuracy of the nomogram.

### 2.11 Collection of Drugs Related to Characteristic Genes and Molecular Docking Verification

For the characteristic genes identified through machine learning, we searched DrugBank to obtain the corresponding related drugs. The 3D structures of the related drugs and characteristic genes were obtained from PubChem (https://pubchem.ncbi.nlm.nih.gov) and the AlphaFold protein structure database (https://alphafold.ebi.ac.uk/). Molecular docking was performed using AutoDock Vina to verify the interactions between the related drugs and characteristic genes.

### 2.12 Statistical Analysis

The molecular docking parameters included an energy range of 5, exhaustiveness of 400, and 20 models to obtain docking binding energies. For GEO file extraction and data annotation, Strawberry Perl 5.32.1.1 was used. Independent sample *t*‐tests were used to compare two independent samples, whereas the Wilcoxon signed‐rank test was used for two paired samples. For data with three or more groups, one‐way analysis of variance (ANOVA) and the Kruskal–Wallis rank‐sum test were utilized. Spearman rank correlation tests were conducted for correlation analysis. All statistical analyses were two‐sided and performed using the “TwoSampleMR” package in R software 4.2.1.

## 3 Results

### 3.1 Two‐Sample MR Analysis Results of Plasma Proteins With IS and VD

**FIGURE 2:**
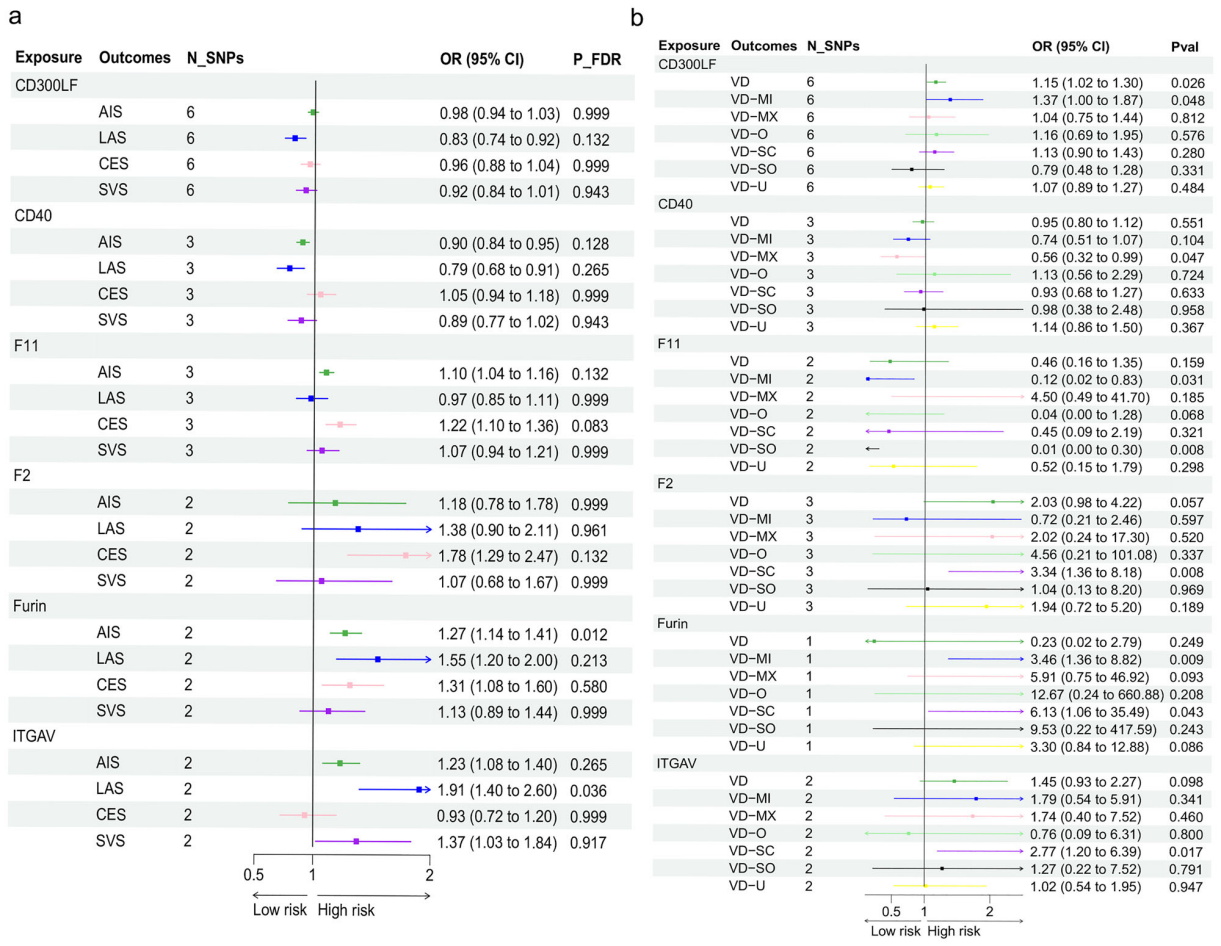
**Six proteins have significant causal relationships with both IS and VD**. AIS, any ischemic stroke; CES, cardioembolic stroke; LAS, large artery stroke; SNPs, single‐nucleotide polymorphisms; SVS, small vessel stroke; VD, vascular dementia; VD‐MI, vascular dementia‐multiple infarctions; VD‐MX, vascular dementia‐mixed; VD‐O, vascular dementia‐other; VD‐SC, vascular dementia‐subcortical; VD‐SO, vascular dementia‐sudden onset; VD‐U, vascular dementia‐undefined. (a) Forest plot of MR estimates of six plasma proteins for four subtypes of IS. (b) Forest plot of MR estimates of the same proteins for five subtypes of VD.

We identified IVs for 1984 *cis*‐plasma proteins. Using the IVW/Wald ratio method, we found 24 *cis*‐plasma proteins associated with IS subtypes and 306 *cis*‐plasma proteins associated with VD subtypes. The intersection of these sets revealed 6 plasma proteins with causal relationships to IS and VD: CMRF35‐like molecule 1 (CD300LF), tumor necrosis factor receptor superfamily member 5 (TNFRSF5) (CD40), coagulation factor XI (FXI) (F11), prothrombin (F2), Furin, and ITGAV. CD40 demonstrated a negative causal relationship with AIS (Figure 2a) and VD‐MX (Figure 2b). Conversely, F2 exhibited a positive causal relationship with CES (Figure 2a) and VD‐SC (Figure 2b). Additionally, Furin increased the risk of AIS by 1.27 times (Figure 2a) and VD by 3.46 times (Figure 2b). ITGAV raised the risk of LAS by 1.91 times (Figure 2a) and VD‐SC by 2.77 times (Figure 2b). These associations were confirmed using alternative MR analysis methods.

### 3.2 SMR Analysis Results of Six Plasma Proteins Associated With IS and VD

The SMR method identified significant associations of six plasma proteins with IS and VD. F11 showed significant positive regulatory relationships with AIS and CES. F2 also had significant positive regulatory relationships with AIS and CES. Furin exhibited significant positive regulatory relationships with AIS, LAS, and CES. CD40 displayed significant negative regulatory relationships with AIS and LAS. Additionally, CD300LF showed a near‐significant positive regulatory relationship with VD, and ITGAV and CD40 displayed near‐significant positive regulatory relationships with VD‐U. F2 had a near‐significant negative regulatory relationship with VD‐MI, whereas CD40 had a near‐significant negative regulatory relationship with VD‐MX. These findings reinforced the robustness of our previous results, and HEIDI analysis indicated no evidence of heterogeneity (Figure S1).

### 3.3 Mediation Effect of Six Plasma Proteins on IS and VD Outcomes via Risk Factors

**FIGURE 3:**
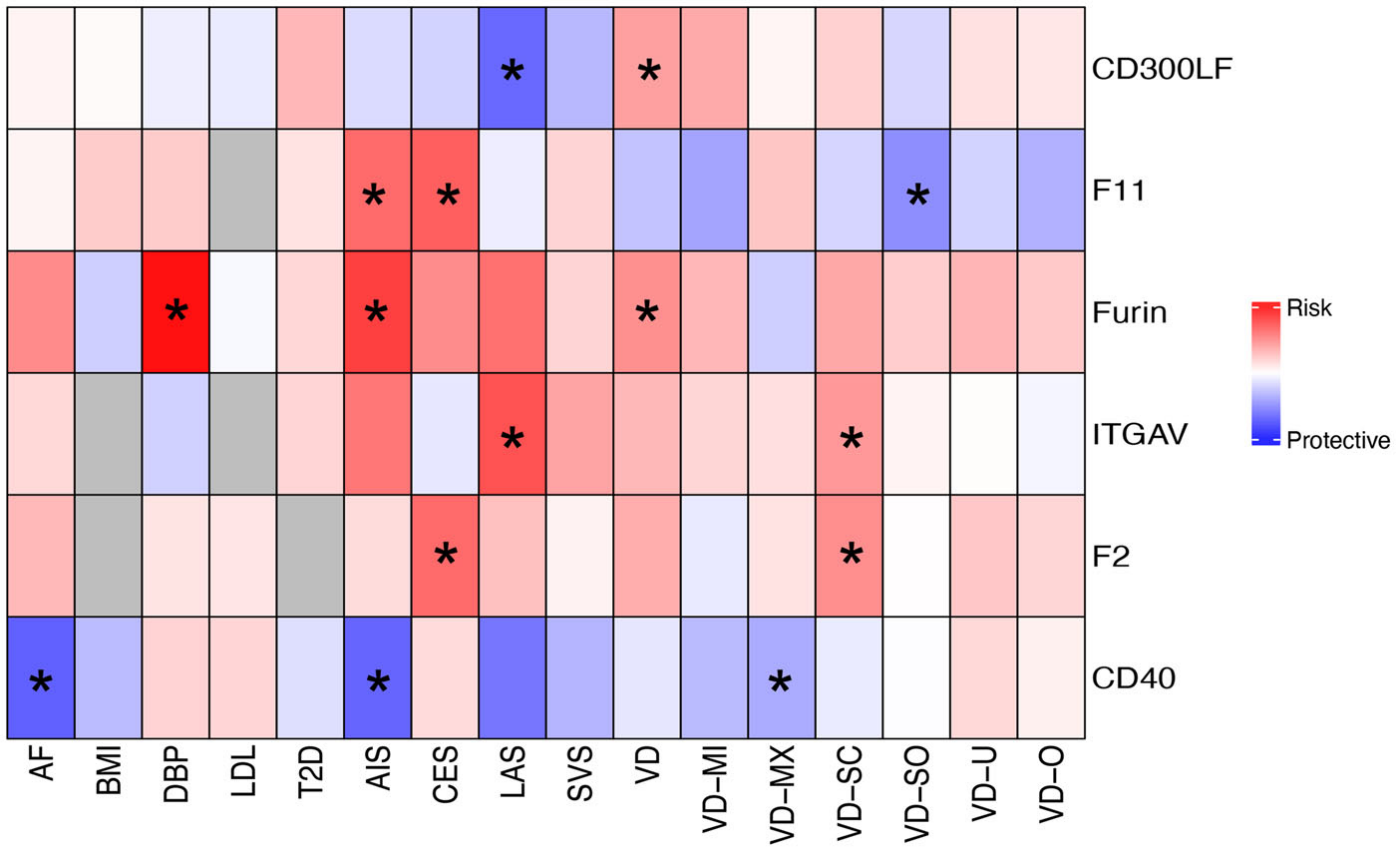
**CD40 and Furin have causal relationships with AF and DBP**. AIS, any ischemic stroke; CES, cardioembolic stroke; LAS, large artery stroke; SVS, small vessel stroke; VD, vascular dementia; VD‐MI, vascular dementia‐multiple infarctions; VD‐MX, vascular dementia‐mixed; VD‐O, vascular dementia‐other; VD‐SC, vascular dementia‐subcortical; VD‐SO, vascular dementia‐sudden onset; VD‐U, vascular dementia‐undefined.

**FIGURE 4:**
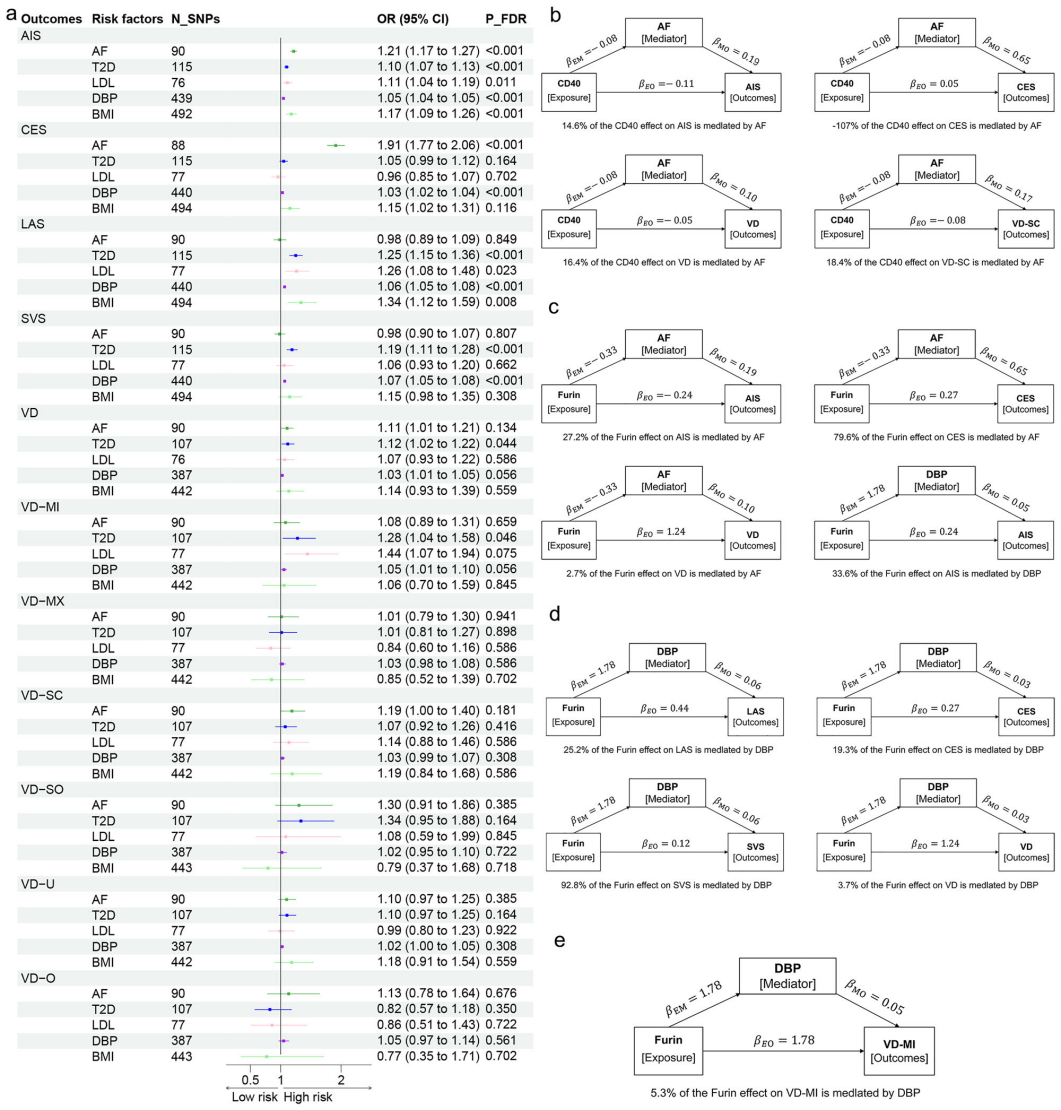
**Mediation effect of six plasma proteins on IS and VD outcomes via AF and DBP**. AF, atrial fibrillation; AIS, any ischemic stroke; BMI, body mass index; CES, cardioembolic stroke; DBP, diastolic blood pressure; LAS, large artery stroke; LDL, low‐density lipoprotein; SVS, small vessel stroke; T2D, type 2 diabetes; VD, vascular dementia; VD‐MI, vascular dementia‐multiple infarctions; VD‐MX, vascular dementia‐mixed; VD‐O, vascular dementia‐other; VD‐SC, vascular dementia‐subcortical; VD‐SO, vascular dementia‐sudden onset; VD‐U, vascular dementia‐undefined. (a) Forest plot of MR estimates for four subtypes of IS, seven subtypes of VD, and five vascular risk factors. (b) Mediation of the CD40 effect on IS and VD subtypes via AF. (c) Mediation of the Furin effect on IS and VD subtypes via AF. (d) Mediation of the Furin effect on IS subtypes and VD via DBP. (e) Mediation of the Furin effect on VD‐MI via DBP.

We performed MR analysis on six *cis*‐plasma proteins and 11 risk factors. Among these proteins, CD40 was negatively associated with AF, whereas Furin was positively associated with DBP (Figure 3). AF, T2D, LDL, and DBP were both associated with IS and VD (Figure 4a, *p*‐FDR < 0.15). Mediation analysis showed that CD40‐mediated AIS through AF with an effect of −0.016 (effect proportion: 14.6%), CES with an effect of −0.053 (effect proportion: −107%), VD with an effect of −0.008 (effect proportion: 16.4%), and VD‐SC subtype with an effect of −0.014 (effect proportion: 18.4%) (Figure 4b). Furin's indirect effect on AIS through AF was 0.065 (effect proportion: 27.2%), on CES it was 0.217 (effect proportion: 79.6%), and on VD it was 0.034 (effect proportion: 2.7%) (Figure 4c). Furin's indirect effect on AIS through DBP was 0.08 (effect proportion: 33.6%) (Figure 4c), on LAS it was 0.11 (effect proportion: 25.2%), on CES it was 0.053 (effect proportion: 19.3%), on SVS it was 0.114 (effect proportion: 92.8%), on VD it was 0.045 (effect proportion: 3.7%) (Figure 4d), and on VD‐MI subtype it was 0.094 (effect proportion: 5.3%) (Figure 4e).

### 3.4 PheWAS Analysis of IS‐ and VD‐Associated Plasma Proteins

To determine whether CD40 and Furin have broader implications, we conducted a PheWAS analysis of 2272 diseases and traits in the FinnGen R9 database. CD40 was associated with an increased risk of rheumatoid arthritis, thyrotoxicosis, and Graves’ disease. Furin was linked to AF and flutter with reimbursement, heart failure, and other heart diseases, aligning with its effects on IS and VD (Figure S2). This suggests that Furin might also be a potential therapeutic target for heart diseases.

### 3.5 In Vivo Experiments Confirmed That Four Plasma Proteins Were Causally Associated With IS and VD

**FIGURE 5:**
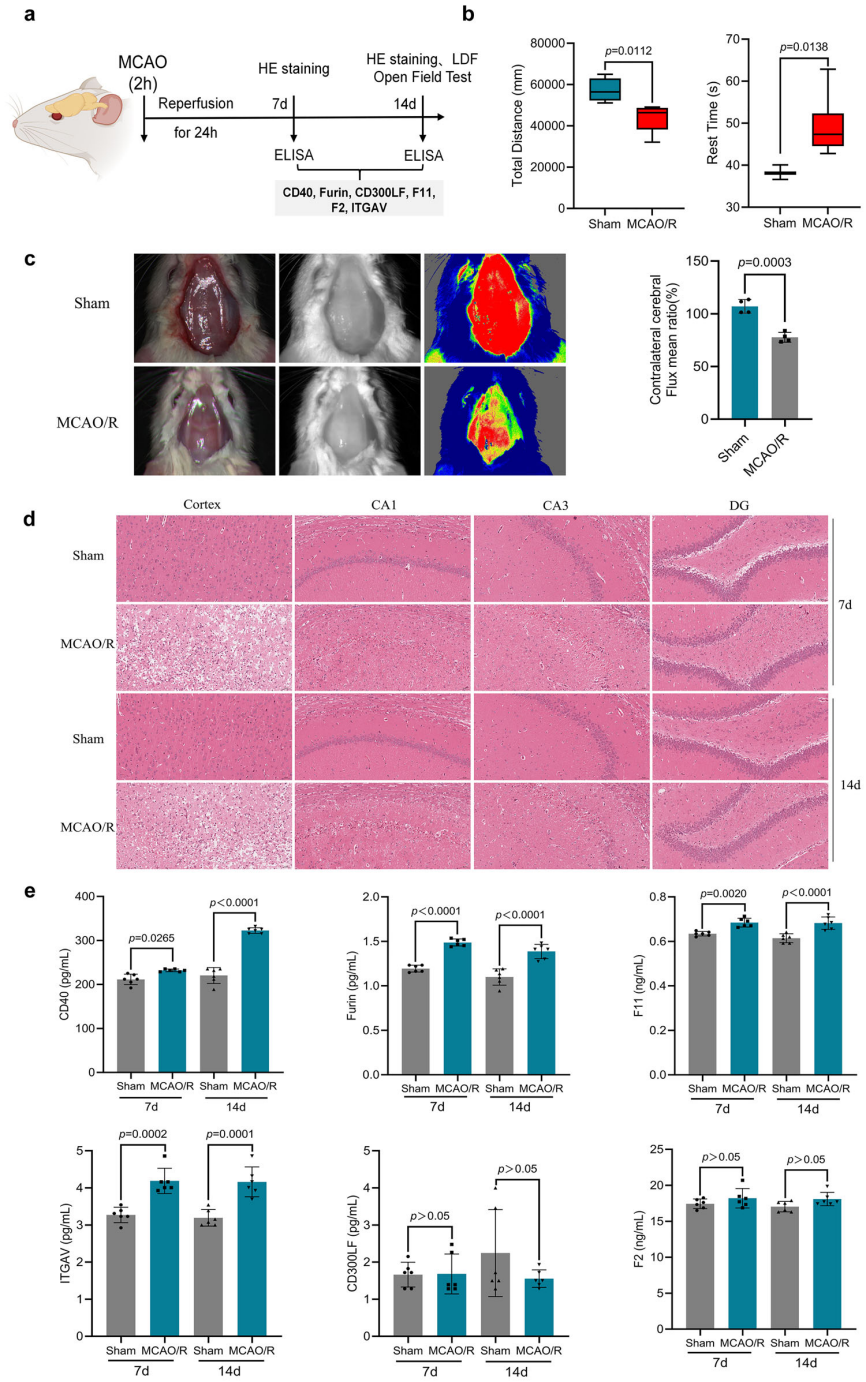
**In vivo experiments confirmed that four plasma proteins were causally associated with IS and VD**. (a) Schematic of the experimental paradigm. (b) Open‐field testing was performed on 14th day. (c) Laser Doppler flowmetry was employed to assess cerebral blood flow distribution in rats on 14th day. (d) Hematoxylin–eosin staining was conducted to examine pathological morphology. Cerebral cortex, CA1, CA3 (20×). The images were visualized with K‐viewer software (scale bar = 50 µm). (e) Serum concentrations of CD40, Furin, CD300LF, F11, F2, and ITGAV were quantified using rat‐specific ELISA kits on Days 7 and 14 post‐modeling.

All rat experiments were conducted using a randomized, double‐blind experimental design (Figure 5a). Open field test results demonstrated that on postoperative Day 14, compared with the Sham group, rats in the model group exhibited significantly reduced total distance and prolonged rest time in the open field (Figure 5b), indicating the successful establishment of the VD animal model. LDF revealed markedly decreased cerebral blood flow in the model group compared to the Sham group on Day 14 post‐surgery (Figure 5c). HE staining showed irregular neuronal morphology, pyknotic nuclei with blurred contours, reduced nuclear counts, and disordered pyramidal cell arrangement in the cortex and hippocampal CA1/CA3 regions of the model group on Days 7 and 14 post‐surgery compared to the Sham group (Figure 5d). These findings suggest that both IS and VD models induced significant neuronal damage, leading to apoptosis and necrosis. ELISA results indicated that serum levels of CD40, Furin, F11, and ITGAV were significantly elevated in the model group compared to the Sham group on Days 7 and 14 post‐surgery, whereas CD300LF and F2 showed no significant changes (Figure 5e). In vivo experiments confirmed the causal associations of CD40, Furin, F11, and ITGAV with IS and VD, which were substantially consistent with MR findings, thereby enhancing the plausibility of the MR results.

### 3.6 PPI Analysis of IS‐ and VD‐Associated Plasma Proteins and Corresponding Gene Expression Analysis

**FIGURE 6:**
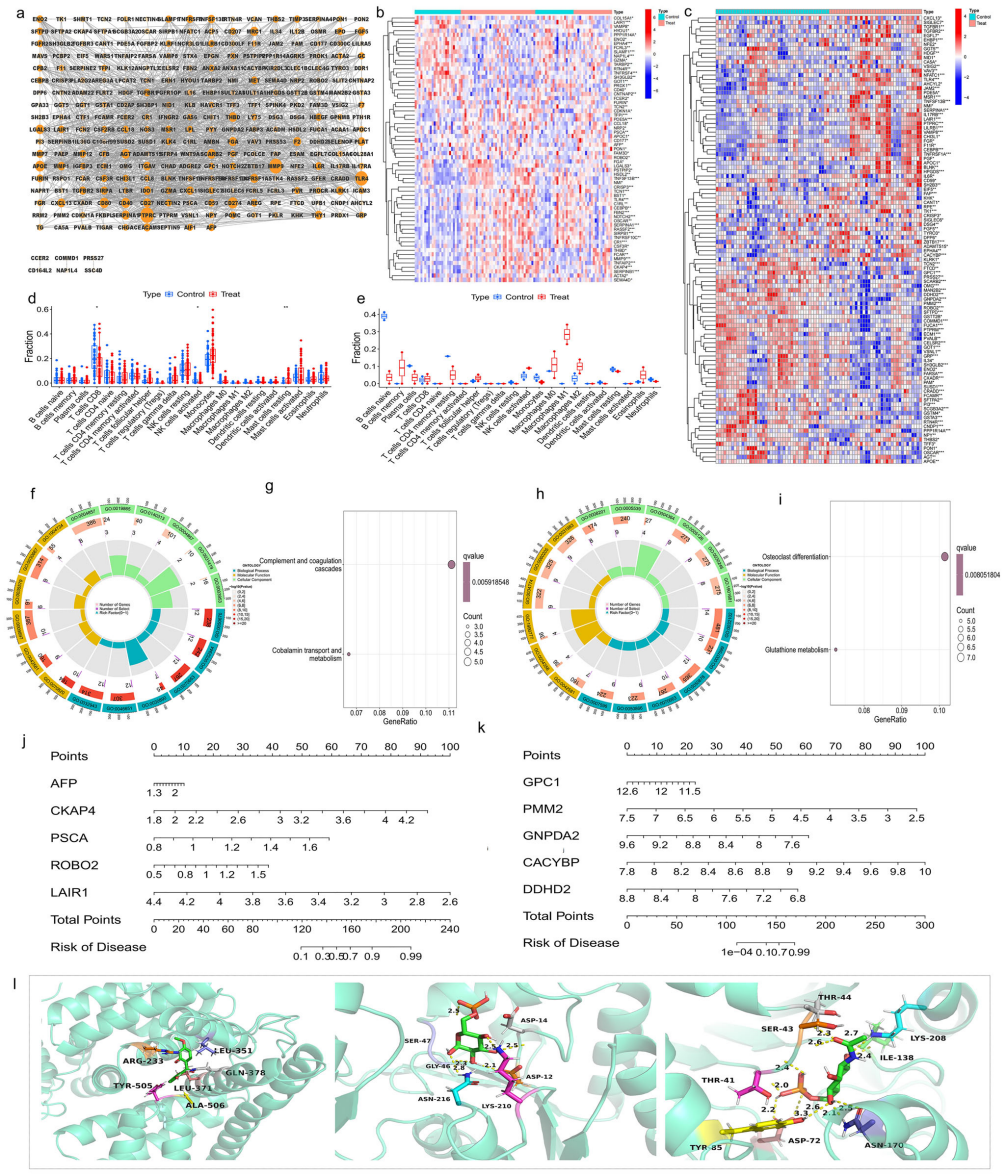
**Results of integrated bioinformatics analyses**. (a) PPI network of plasma proteins associated with IS and VD. (b) Heatmap of the differential expression of IS‐ and VD‐associated targets between IS and control groups. (c) Heatmap of the differential expression of IS‐ and VD‐associated targets between VD and control groups. (d) Boxplot of immune cell differential expression analysis between IS and control groups. (e) Boxplot of immune cell differential expression analysis between VD and control groups. (f) Circular plot of GO enrichment analysis for DEGs in IS samples. (g) Bubble plot of KEGG pathway enrichment analysis for DEGs in IS samples. (h) Circular plot of GO enrichment analysis for DEGs in VD samples. (i) Bubble plot of KEGG pathway enrichment analysis for DEGs in VD samples. (j) Nomogram of IS characteristic genes constructed using the XGB machine learning method. (k) Nomogram of VD characteristic genes constructed using the SVM machine learning method. (l) Conceptual illustration of molecular docking between the best‐performing drug components and the positive targets.

We imported 24 IS‐associated and 306 VD‐associated plasma proteins into the STRING database for PPI analysis. This resulted in a network comprising 287 nodes and 1349 edges, with interactions among proteins such as ACAA1, ACADM, ACP5, ACTA2, and ADAM22 (Figure 6a). Differential expression analysis revealed that 63 genes, including CD40, Furin, ACTA2, AFP, APOC1, BST1, and C1RL, were differentially expressed between the IS and control groups (Figure 6b). Similarly, 102 genes, including ADAMTS15, AGT, AHCYL2, APOC1, and APOE, showed differential expression between the VD and control groups (Figure 6c). Correlation analysis of DEGs in IS and VD samples indicated strong, primarily negative correlations (Figures S3a and 3b).

### 3.7 Immune Cell Infiltration, Differential Expression, Correlation, and DEGs Enrichment Analysis

We conducted immune cell infiltration analysis to determine the types and quantities of immune cells in each sample, employing ssGSEA to identify significant immune cells between control and IS or VD groups. Notably, T cells, CD, and NK cells were highly expressed in the control group, whereas resting mast cells were predominant in the IS group (Figure 6d). No statistically significant differences in immune cell expression were observed in VD samples (Figure 6e). Correlation analysis showed positive correlations between DEGs and immune cells in IS samples (Figure S3c), but no significant correlations in VD samples (Figure S3d).

GO and KEGG pathway enrichment analyses were performed on DEGs from IS and VD samples. BP for IS DEGs included lymphocyte proliferation regulation, and MF included enzyme inhibitor activity and immunoglobulin binding (Figure 6f). KEGG pathways encompassed complement and coagulation cascades, and cobalamin transport and metabolism (Figure 6g). For VD DEGs, enriched BP included hemostasis and body fluid regulation, and MF included glutathione transferase activity (Figure 6h). KEGG pathways involved glutathione metabolism and osteoclast differentiation (Figure 6i).

### 3.8 Machine Learning Prediction Model for IS and VD

We developed four machine learning models (SVM, RF, XGB, and GLM) using DEG data for IS and VD. Analysis of ROC curves, residual box plots, and inverse cumulative distribution plots identified the XGB model for IS and the SVM model for VD as the most accurate, evidenced by the highest area under the ROC curve (AUC) and the lowest residuals and inverse cumulative values (Figure S4a–h). These models were used to calculate importance scores for characteristic genes, leading to the creation of nomograms for the top five genes in the IS model (AFP, CKAP4, PSCA, ROBO2, and LAIR1) and the VD model (GPC1, PMM2, GNPDA2, CACYBP, and DDHD2), with corresponding scoring scales (Figure 6j,k). By calculating the total expression scores of these characteristic genes, we predicted the risk rates of IS and VD characteristic genes in the development of IS and VD, thereby determining treatment sensitivity. Prediction accuracy was confirmed by the close alignment of solid and dashed lines in the calibration curves (Figure S5a,b) and the proximity of red and gray lines in the decision curves (Figure S5c,d).

### 3.9 Drug Collection and Molecular Docking Validation for Characteristic Genes

Using the DrugBank database, we identified drugs targeting genes such as AFP, PSCA, GNPDA2, PMM2, and CACYBP associated with IS and VD. For targets with available 3D structures, molecular docking was performed using AutoDock Vina. Except for the CACYBP‐calcium citrate combination, all docking results indicated potential for stable structure formation (Table S1). Visualization of the best docking results revealed positive target–drug interactions, such as AFP‐dioxybenzone, GNPDA2‐*N*‐acetyl‐d‐glucosamine‐6‐phosphate, and PMM2‐alpha‐d‐glucose 6‐phosphate, primarily mediated through hydrogen bonds and hydrophobic pockets (Figure 6l).

## 4 Discussion

This study is the first large‐sample investigation to systematically identify potential targets for IS and VD using human plasma proteins GWAS data. On the basis of GWAS summary data of 2923 plasma proteins, our research provides robust evidence that six proteins (CD300LF, CD40, F11, F2, Furin, and ITGAV) are causally associated with IS and VD. CD300LF exhibits a bidirectional regulatory effect, reducing the risk of IS while increasing the risk of VD. CD40 can decrease the risk of both IS and VD. F11 acts as a risk factor for IS but reduces the risk of VD. F2 increases the risk of both IS and VD. Furin and ITGAV similarly elevate the risk of both conditions. SMR analysis validated that four of these proteins (CD40, F11, F2, and Furin) were consistent with the primary findings, demonstrating the robustness of our results. We found that AF, BMI, DBP, T2D, and LDL are causally associated with the risk of various IS subtypes, consistent with previous studies and classical epidemiological data (Chen et al. 2022; Yang et al. 2019; Mitchell et al. 2015; Kivimäki et al. 2017; Janghorbani et al. 2007). Our analysis also revealed that AF, DBP, T2D, and LDL are linked to the risk of VD, highlighting their crucial role in VD pathogenesis (Gottesman and Seshadri 2022). Age and APOE genotype remain the most significant risk factors for brain health and VD (Liu et al. 2013). Our findings suggest that many IS risk factors also elevate VD risk, offering new insights for VD prevention. Mediation analysis revealed that CD40 mediates the effects on IS subtypes (CES, AIS), VD, and the VD‐SC subtype by regulating AF. Furin mediates the effects on IS subtypes (CES, AIS, and CES) by regulating AF. Additionally, Furin mediates the risk of IS subtypes (AIS, LAS, and SVS) and VD by regulating DBP. PheWAS analysis indicated that CD40 and Furin have detrimental effects on rheumatoid arthritis, AF, and other heart diseases. Furin's impact on AF and other heart conditions aligns with its effects on IS and VD, suggesting that Furin could be a common therapeutic target for IS and VD and heart diseases like AF. In vivo experiments confirmed the causal associations of CD40, Furin, F11, and ITGAV with IS and VD, further validating the potential of these plasma proteins as therapeutic targets for IS and VD.

CD300LF, an important receptor on immune cells, plays a key role in regulating immune responses. It is expressed on monocytes and microglia, crucial for managing inflammatory responses and immune cell activity. Studies indicate that CD300LF regulates microglial activation, reduces demyelination, and lowers inflammatory factor release, potentially protecting against IS‐related inflammation (Choi et al. 2011). Its presence in infiltrating monocytes/macrophages suggests that it may have a potential role in neuroinflammation and neuroprotection (Ejarque‐Ortiz et al. 2015). In models of penetrating brain injury, CD300LF reduces neuroinflammation and supports neuron survival, indicating a protective role in brain injury repair processes (Alí‐Ruiz et al. 2023). These studies support the protective effects of CD300LF on IS observed in our study. Conversely, CD300LF may contribute to the chronic inflammatory state of VD by regulating inflammatory responses and immune cell activity, highlighting its potential impact on neurodegeneration and cognitive decline (Cao et al. 2021; Elgueta et al. 2009). This supports our finding that CD300LF increases the risk of VD. Research mainly focuses on how CD300LF influences IS and VD progression by regulating neuroinflammatory responses and immune cell functions, suggesting its potential as a therapeutic target for IS and VD.

CD40, also known as TNFRSF5, is a crucial cell surface receptor within the tumor necrosis factor (TNF) receptor superfamily (Ots et al. 2022). CD40 and its ligand CD40L are pivotal in various neurological disorders, including traumatic brain injury, Alzheimer's disease, Parkinson's disease, stroke, epilepsy, nerve injury, and multiple sclerosis (Chen et al. 2022). Our research indicates that CD40 reduces the risk of AIS, corroborating prior findings (Emsley et al. 2010). Moreover, CD40 diminishes the risk of mixed subtype VD. F11 is an enzyme essential for the blood coagulation process, specifically activating FXI during the amplification phase of the coagulation cascade (Leung et al. 2012). F11 plays a significant role in the pathophysiology of IS by facilitating thrombus formation and propagation (Salomon et al. 2008). Elevated F11 activity is correlated with an increased stroke risk, positioning F11 as a potential target for IS prevention and treatment (Shoamanesh et al. 2022; Gailani and Gruber 2024; Hanson et al. 2013). SNPs in the F11 gene are associated with a heightened risk of IS, particularly in individuals younger than 70 years (Zamolodchikov et al. 2016). Our study confirmed that F11 elevates the risk of IS subtypes (AIS, CES). Notably, we observed that F11 serves as a protective factor for the VD‐MI and VD‐SO subtypes, which contrasts with previous research findings (Brennan and Ahmed 2012). These insights suggest novel preventive and therapeutic strategies targeting F11 for IS, although its impact on VD necessitates further investigation.

Prothrombin, encoded by the F2 gene, is a vital plasma protein in the coagulation cascade (Davie et al. 1991). Synthesized in the liver and converted into active thrombin with the assistance of vitamin K, prothrombin is also produced by neurons and astrocytes (Dihanich et al. 1991; Weinstein et al. 1995). Thrombin plays a central role in blood coagulation by catalyzing the conversion of fibrinogen to fibrin, forming blood clots. Intravascular thrombin and the coagulation cascade have been longstanding targets for IS treatment, involving the use of warfarin, platelet inhibitors, and direct thrombin inhibitors (Ye et al. 2021). Elevated thrombin levels are detected in infarct areas post‐ischemia due to blood–brain barrier disruption and cerebral prothrombin synthesis (Chen et al. 2012; Bushi et al. 2013). Multiple research groups have studied the role of thrombin in cerebral ischemia using in vivo and in vivo models. High thrombin concentrations activate microglia and induce astrocyte and neuron death, whereas low concentrations have neuroprotective effects in cerebral ischemia (Xi et al. 2003). Therefore, elevated prothrombin levels increase the risk of neuroinflammatory diseases, including IS and VD. Furin, ubiquitously present in various cell types, is integral to the maturation of proteins and peptides. Neuronal cell death following IS is associated with Furin substrates such as MT1‐MMP, hepcidin, and angiogenin (Wichaiyo et al. 2024). Inhibition of Furin can mitigate vascular remodeling and atherosclerosis, supporting the potential for Furin‐targeted interventions to reduce the risk of IS and VD (Yakala et al. 2019).

The identification of proteins positive for both IS and VD by intersecting IS‐ and VD‐related proteins yielded a relatively conservative result, which can be supplemented by the protein interaction network. GEO human transcriptome data showed that two out of the six intersecting proteins (CD40, Furin) are differentially expressed between IS and normal groups, whereas none of the six intersecting proteins showed differential expression between VD and normal groups. Consequently, we further screened IS and VD proteins related to the PPI network to obtain DEGs and conducted multiple analyses on the basis of DEGs, including those involving immune cells and pathways. The results indicated that it involves immune cells, consistent with previous studies. However, VD did not show positive results, which may be due to the fact that VD samples were from autopsies and had a small sample size. The DEGs of IS were enriched in the complement and coagulation cascade reactions and cobalamin transport and metabolism, both of which are protective factors for IS (Maners et al. 2020; Roth and Mohamadzadeh 2021). The DEGs of VD were enriched in the glutathione metabolism pathway. Previous studies have shown that glutathione plays a critical role in protecting neurons from oxidative stress, which is a primary mechanism in VD occurrence post‐IS (Higashi et al. 2021). Through machine learning analysis of DEGs, we identified 10 feature genes related to IS and VD, capable of predicting the onset of IS or VD. Most of the drugs identified through these feature genes can form stable structures, making them potential therapeutic candidates.

Our study has some limitations. First, when screening causally related proteins for IS and VD, although we preset the random effects IVW model as the main analysis result, during the screening process for VD, we included two proteins (CD40, Furin) selected by the weighted median and Wald ratio methods. Both CD40 and Furin were selected under the IVW model during the IS screening process. Given the relatively few cases of VD, we decided to include CD40 and Furin as positive screening results for further analysis to broaden the scope. Additionally, we performed multi‐trait colocalization analysis and found that the associations between CD40 and LAS, Furin and AIS, and ITGAV and LAS were due to the same genetic locus. Studies have shown that a lack of colocalization evidence does not invalidate research results, as colocalization methods have a high false‐negative rate (usually around 60%) (Hukku et al. 2021). Therefore, even though our multi‐trait colocalization analysis only identified some positive proteins consistent with the MR analysis, this does not indicate poor robustness of our results. Circulating plasma proteins do not fully represent protein changes in the central nervous system (CNS), whereas CSF often provides a more direct reflection of brain pathophysiological processes. Although our findings are considered a preliminary step in target screening rather than conclusive evidence for drug targets, future research should combine CSF proteomics and brain tissue data to validate and refine candidate targets identified from plasma discoveries. Moreover, public transcriptomic datasets typically include only limited covariates (e.g., age/sex), precluding parallel incorporation of conventional risk factors; accordingly, we confined the GEO component to independent validation and mechanistic interpretation and will integrate multi‐domain molecular and clinical features in future prospective cohorts.

We advocate for future research to rigorously evaluate the feasibility of these six proteins as therapeutic targets for IS and VD through well‐designed, large‐scale, prospective cohort studies. As the population of individuals with IS and VD grows, the systematic exploration of potential drug targets for elderly multimorbidity becomes increasingly important. Comprehensive proteomic studies on different tissues and organs in the elderly multimorbidity population are essential to evaluate the roles of tissue‐ or organ‐specific proteins in the prevention and treatment of multimorbidity.

## 5 Conclusion

Our study is the first to identify six human plasma proteins with causal relationships to IS and VD, along with their risk factors. In vivo experiments confirmed that CD40, Furin, F11, and ITGAV were causally associated with IS and VD. We investigated the mediating effects of CD40 and Furin on IS and VD through AF or DBP, and their impacts on related conditions. These findings provide new insights into the prevention and treatment of IS and VD using these six plasma proteins as biomarkers. Through integrated bioinformatics analyses of human transcriptome data, we elucidated the mechanisms of action of IS‐ and VD‐related proteins and utilized machine learning to predict the onset of these conditions. Additionally, we identified potential therapeutic drugs capable of forming stable structures with the screened characteristic genes.

## Author Contributions

Jian Lyu, Guibo Sun, and Yanming Xie are responsible for conceptualization, funding acquisition, data curation, investigation, formal analysis, methodology, project administration, software, and visualization. Jian Lyu and Fa Lin are responsible for writing – original draft. Yan Chai looked after writing – review and editing. Fa Lin, Yi Liu, Min Wang, Xiaolin Chen, Fumei Liu, and Haiyan Xiao supervised data curation, formal analysis, methodology, software, and visualization. Jian Lyu and Yanming Xie are the guarantors of this work and, as such, had full access to all the data in the study and take responsibility for the integrity of the data and the accuracy of the data analysis. All authors contributed to the interpretation of the results and critically reviewed the manuscript. All authors read and approved the final version of the manuscript.

## Funding

This work was supported by grants from the National Natural Science Foundation of China (82205101), Fundamental Research Funds for the Central public welfare research institutes (ZZ15‐YQ‐016), Special Fund for Health Development Research of the Capital (2024‐4‐4176), National Key Research and Development Program of China (2018YFC1707400), Special Research Fund of the National Research Center for Traditional Chinese Medicine in Cardiovascular Diseases (CMC2022012), National Science and Technology Major Project for the Prevention and Treatment of Cancer, Cardiovascular and Cerebrovascular, Respiratory and Metabolic Diseases (2024ZD0522100, 2024ZD0522106), and Hospital capability enhancement project of Xiyuan Hospital, CACMS (XYZX0404‐11).

## Conflicts of Interest

The authors declare no conflicts of interest.

## Supporting information



Supplementary Fig. S1–S5

Supplemental information can be found online at: XXXXX.

Supplementary Fig. S1–S5

Supplementary Fig. S1–S5

Supplementary Fig. S1–S5

Supplementary Table S1

## Data Availability

The datasets used in this study are publicly available summary datasets and can be found in cited papers, in the IEU OpenGWAS Project repository (https://gwas.mrcieu.ac.uk/), in the GWAS Catalogue repository (http://ftp.ebi.ac.uk/pub/databases/gwas/summary_statistics/), in the UK Biobank website (https://biobank.ndph.ox.ac.uk/showcase/search.cgi), or in the GEO database (https://www.ncbi.nlm.nih.gov/geo/). There are no restrictions on data availability other than those imposed by the corresponding data committee.
