# Supplementary figures and images for: Identification of Six Potential Therapeutic Targets Common to Ischemic Stroke and Vascular Dementia: Genetic Insights From an Integrated Bioinformatics Analysis
Source: Brain Behav. 2025 Nov 25;15(12):e71096. doi: 10.1002/brb3.71096 (PMC12647924; doi:10.1002/brb3.71096)

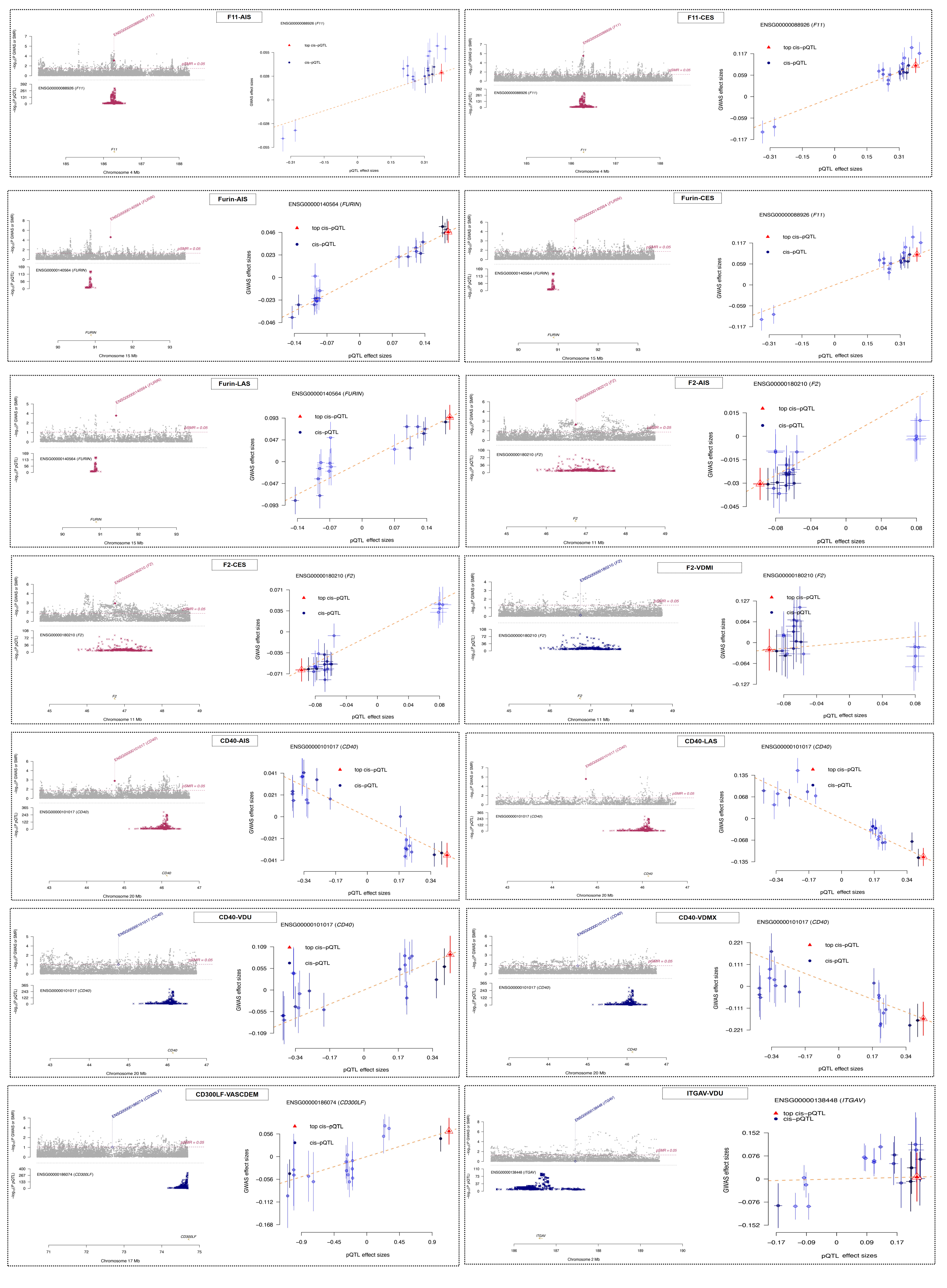

Supplement: Supplementary file 1 — Supplementary Fig. S1–S5 [file BRB3-15-e71096-s002.png]

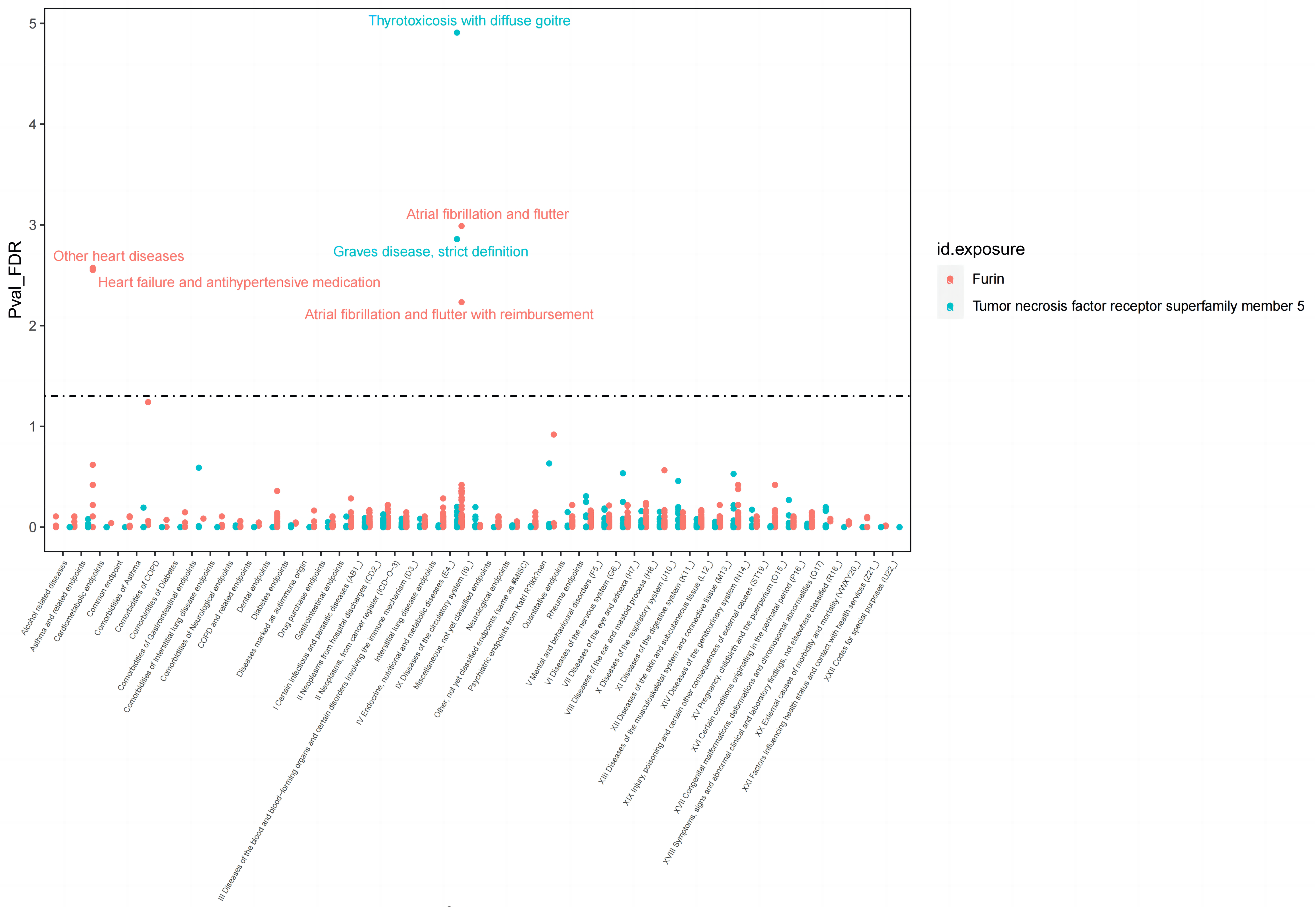

Supplement: Supplementary file 2 — Supplemental information can be found online at: XXXXX. [file BRB3-15-e71096-s003.png]

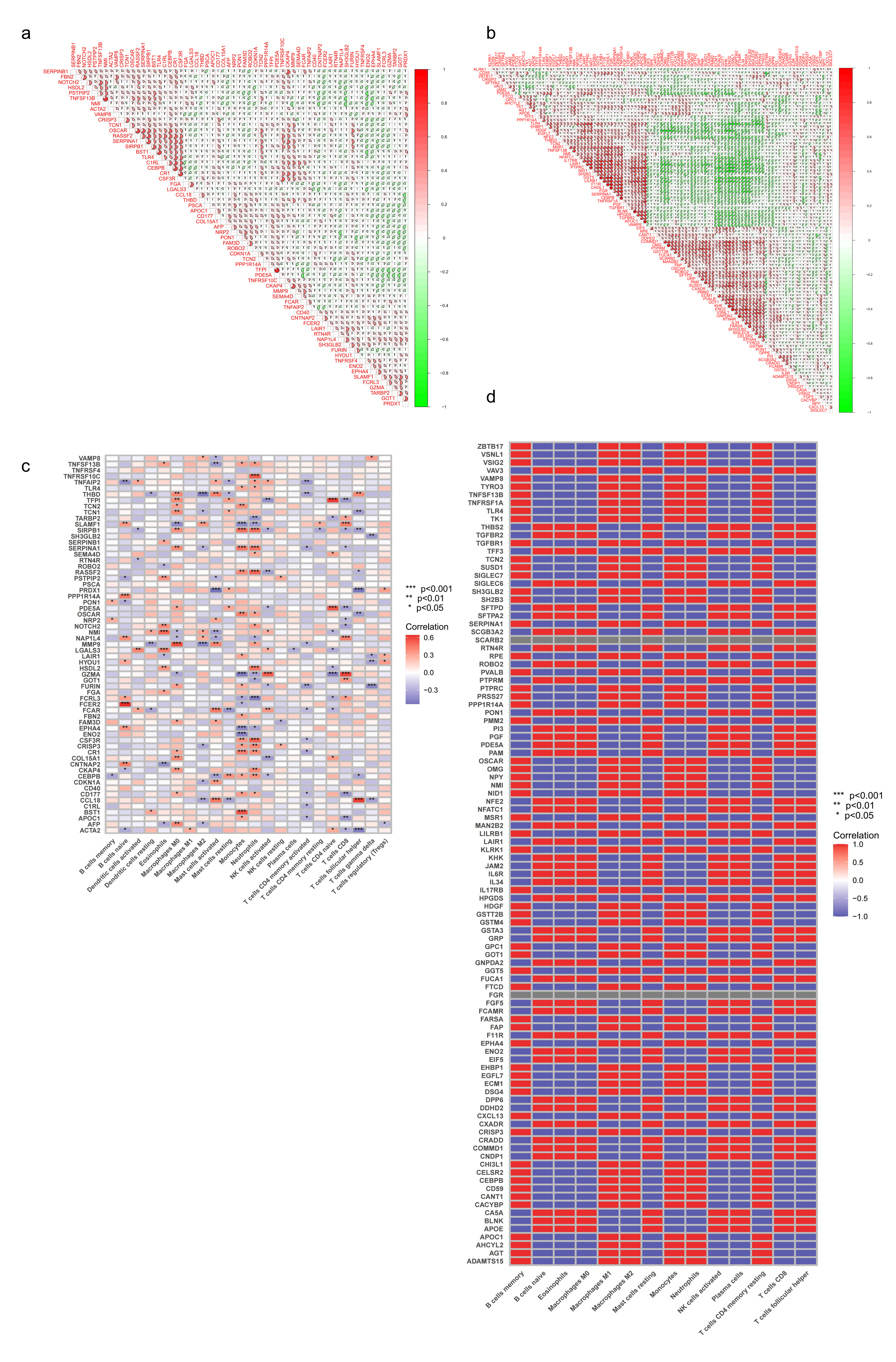

Supplement: Supplementary file 3 — Supplementary Fig. S1–S5 [file BRB3-15-e71096-s005.png]

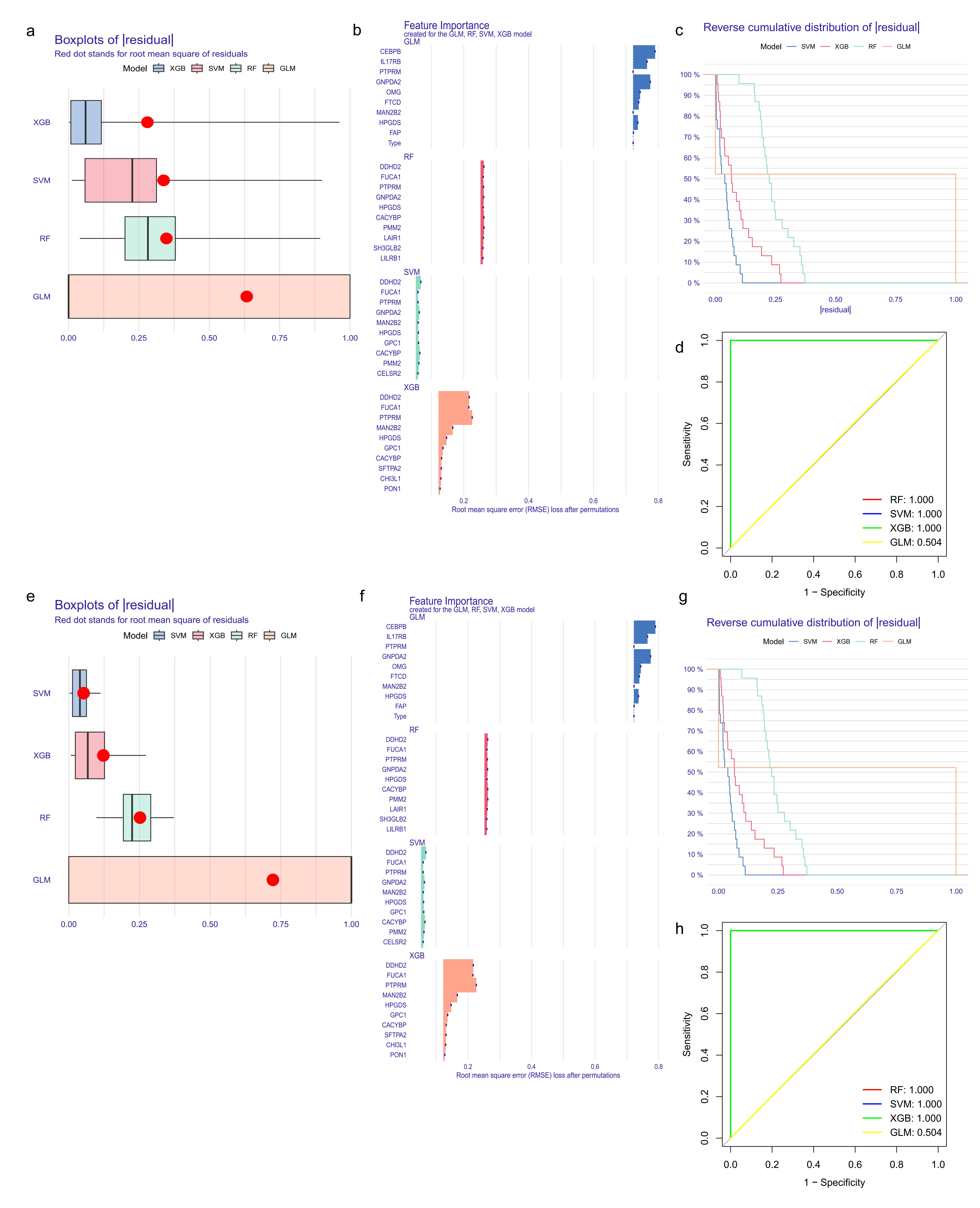

Supplement: Supplementary file 4 — Supplementary Fig. S1–S5 [file BRB3-15-e71096-s006.png]

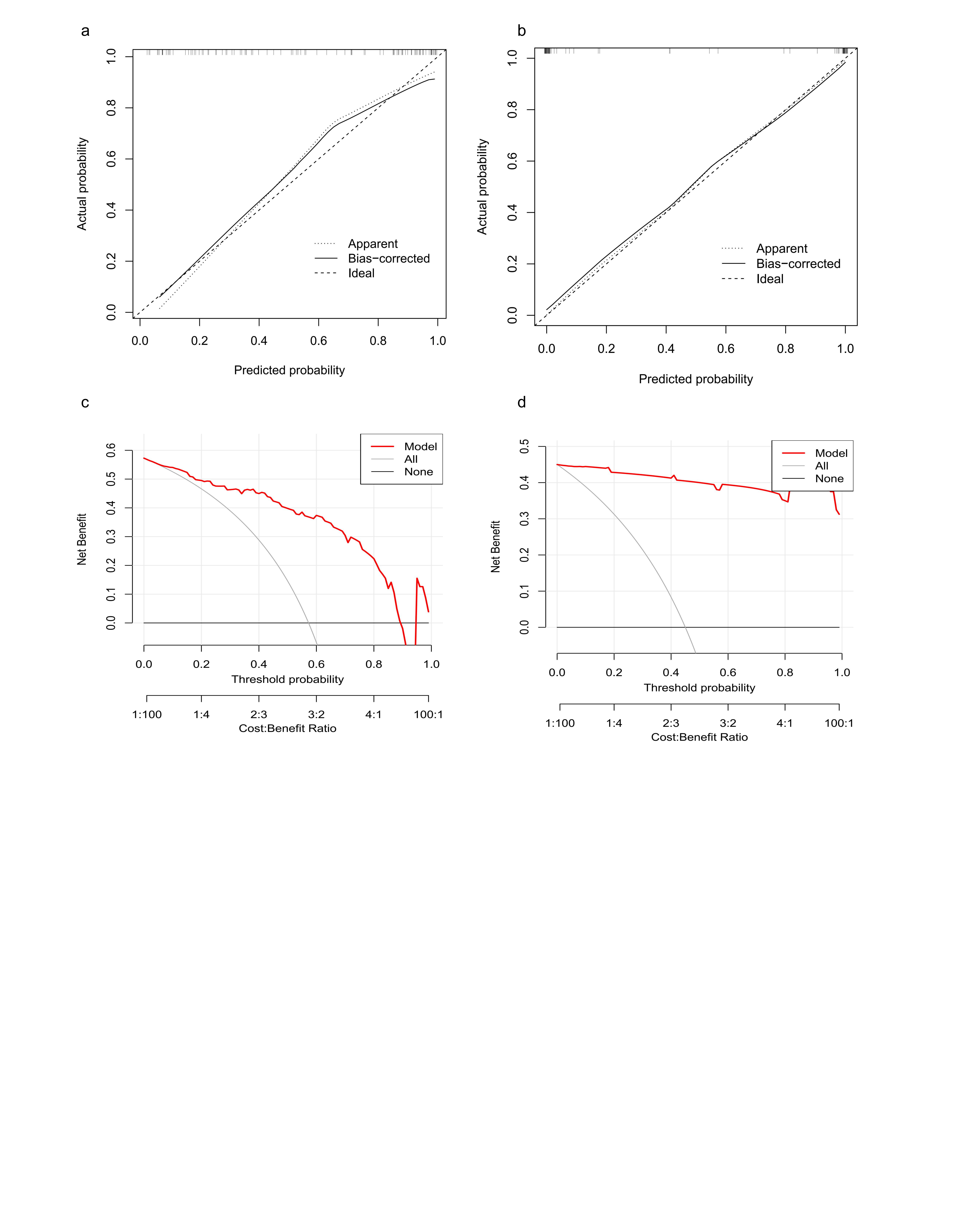

Supplement: Supplementary file 5 — Supplementary Fig. S1–S5 [file BRB3-15-e71096-s004.png]
