# Supplementary material for: Identification of Six Potential Therapeutic Targets Common to Ischemic Stroke and Vascular Dementia: Genetic Insights From an Integrated Bioinformatics Analysis
Source: Brain Behav. 2025 Nov 25;15(12):e71096. doi: 10.1002/brb3.71096 (PMC12647924; doi:10.1002/brb3.71096)
Supplement: Supplementary file 6 — Supplementary Table S1 [file BRB3-15-e71096-s001.doc]

| **Supplementary Table S1 Characteristic gene-related drug molecule docking results** | |
| --- | --- |
| Target | Drug |
| AFP | Dioxybenzone |
| GNPDA2 | 2-Deoxy-2-Amino Glucitol-6-Phosphate |
| GNPDA2 | N-acetyl-D-glucosamine-6-phosphate |
| GNPDA2 | Beta-D-Glucose |
| PMM2 | alpha-D-glucose 6-phosphate |
| PMM2 | alpha-D-glucose-1-phosphate |
| PMM2 | D-Mannose 1-phosphate |
| PMM2 | alpha-D-mannose 6-phosphate |
| PMM2 | Dexfosfoserine |
| CACYBP | Calcium citrate |
